# A cylindrical specimen holder for electron cryo-tomography

**DOI:** 10.1016/j.ultramic.2013.10.016

**Published:** 2014-02

**Authors:** Colin M. Palmer, Jan Löwe

**Affiliations:** MRC Laboratory of Molecular Biology, Cambridge Biomedical Campus, Francis Crick Avenue, Cambridge CB2 0QH, UK

**Keywords:** Electron cryo-tomography, Cryoelectron tomography, Cylindrical specimen, Rod-shaped specimen, Missing wedge, Carbon nanopipettes

## Abstract

The use of slab-like flat specimens for electron cryo-tomography restricts the range of viewing angles that can be used. This leads to the “missing wedge” problem, which causes artefacts and anisotropic resolution in reconstructed tomograms. Cylindrical specimens provide a way to eliminate the problem, since they allow imaging from a full range of viewing angles around the tilt axis. Such specimens have been used before for tomography of radiation-insensitive samples at room temperature, but never for frozen-hydrated specimens. Here, we demonstrate the use of thin-walled carbon tubes as specimen holders, allowing the preparation of cylindrical frozen-hydrated samples of ribosomes, liposomes and whole bacterial cells. Images acquired from these cylinders have equal quality at all viewing angles, and the accessible tilt range is restricted only by the physical limits of the microscope. Tomographic reconstructions of these specimens demonstrate that the effects of the missing wedge are substantially reduced, and could be completely eliminated if a full tilt range was used. The overall quality of these tomograms is still lower than that obtained by existing methods, but improvements are likely in future.

## Highlights

• The missing wedge is a serious problem for electron cryo-tomography.
• Cylindrical specimens allow the missing wedge to be eliminated.
• Carbon nanopipettes can be used as cylindrical holders for tomography of frozen-hydrated specimens.
• Cryo-tomography of cylindrical biological samples demonstrates a reduction of deleterious effects associated with the missing wedge.

## 1 Introduction and theory

Electron cryo-tomography (cryo-ET) is a three-dimensional imaging technique capable of visualising unstained biological material in a near-native state at a resolution of a few nanometres. Cryo-ET has rapidly increased in popularity in recent years, and many reviews are now available [1–5]. The technique has produced some highly significant results [6–9], but two major limitations currently restrict the quality of reconstructions generated by cryo-ET.

First, unstained biological specimens are easily damaged by the electron beam, and have low inherent contrast in the TEM, producing images with a low signal-to-noise ratio (S/N). Higher electron doses improve the S/N, but also damage the specimen, resulting in a compromise between the electron dose and the attainable resolution [10,11]. The S/N is reduced further by the use of thick specimens, so samples must be kept as thin as possible (well under 1 µm) for best results. Various technological improvements are currently being developed in an attempt to improve the situation, including direct electron detectors to improve the S/N of individual images [12,13], phase plates to increase image contrast [14], and cryo-sectioning and focussed ion beam milling to reduce specimen thickness [15–18].

The second major limitation that affects cryo-ET is the limited range of angular sampling imposed by the use of extended flat specimens, prepared on standard TEM grids. When a slab-like flat specimen is tilted for tomography, the effective specimen thickness in the electron beam increases, decreasing the S/N in the images. At high tilt angles, this effect becomes severe and images provide no useful signal above the background noise. After a tomogram is reconstructed, a wedge-shaped region in Fourier space is left containing no information, and this is named the “missing wedge” problem.

**Fig. 1:**
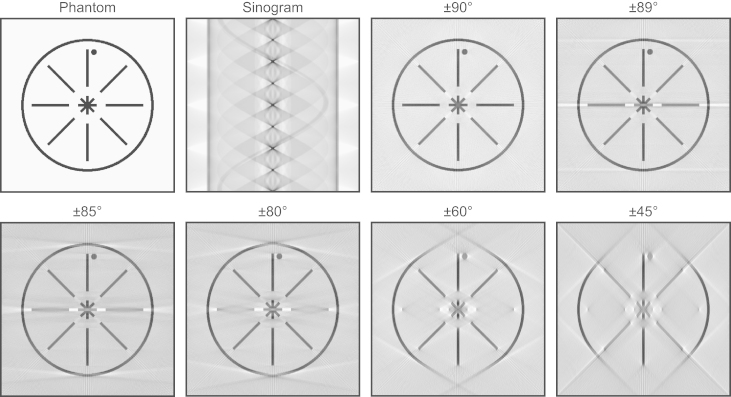
Simulation of the effects of the missing wedge. A phantom image was projected at 1° intervals to produce the sinogram shown. A reconstruction from a full 180° range of angles (±90°) shows some loss of contrast and minor artefacts due to the tilt increment, but is otherwise an accurate copy of the original phantom. As the range of angles used for reconstruction is reduced, more serious artefacts appear and horizontally-oriented structures are almost completely lost from the images.

**Fig. 2:**
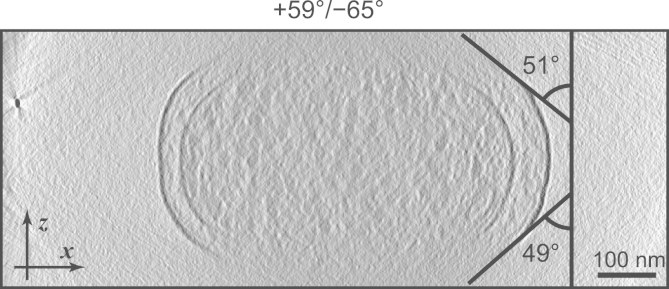
A real example of the effects of the missing wedge. The image shows a cross-section from a reconstruction of a frozen *Escherichia coli* cell, with a gold fiducial marker visible at the extreme left. The cell membranes are only resolved at the sides of the cell, and white streaking artefacts are visible around the gold particle. The black lines show tangents to the outer cell membrane and their angles from the *z* axis. The angular range of the tilt series used for this tomogram is shown above the image.

The missing wedge causes several deleterious effects in tomograms. These are demonstrated in the simulation presented in Fig. 1 and can also be seen in the real example shown in Fig. 2. Most strikingly, horizontally-oriented features disappear almost entirely as the size of the missing wedge increases. In Fig. 1, this effect can be seen most clearly on the horizontal lines and the large circle, but also on the horizontal part of the central star shape, showing that horizontally-oriented parts of more complex structures are also lost. In Fig. 2, the cell membranes are obviously incomplete, and the ice layer that contains the cell is entirely missing from the reconstruction, since its surfaces are horizontal and therefore aligned with the missing wedge. Compact objects – such as the small circle in Fig. 1 and the ribosomes in Fig. 2 – are not lost completely, but do suffer from elongation in the direction of the missing wedge, reducing the resolution in this direction. This elongation has been expressed as a multiplication factor by Radermacher [19], describing the lengthening of an ellipsoidal point spread function (PSF) along the *z* axis. However, this treatment does not explain the complete loss of extended horizontal structures from the reconstruction, and it seems the full effect of the missing wedge cannot simply be explained by convolution of an ellipsoidal PSF with the points composing the object.

Another effect of the missing wedge is the presence of white halos and streaking artefacts that appear at a tangent from objects with high contrast (most features in Fig. 1, and the gold fiducial marker on the left of Fig. 2). It is notable that curved linear structures, such as the large circle in Fig. 1, are visible only between these tangents, and their angles match the size of the missing wedge. This is instructive when observing real tomography data: the tangent angles where the cell membranes fade away can give an indication of the angular range where useful information is obtained from the images. These angles are often measured to be lower than the full range that was used for image collection. In the example shown in Fig. 2, tangents to the last clearly-visible parts of the cell membrane have angles of +49° and −51°, even though images were collected over a much larger range of +59° to −65°. This suggests that the images from higher tilt angles contribute little or no information to the reconstructed tomogram (due to the increased thickness at high tilt and the resulting low signal-to-noise ratio in the images) and so the missing wedge is larger than expected. This could have some relevance in situations where the size of the missing wedge is used in subsequent calculations, such as the estimation of resolution or the masking of objects for sub-tomogram averaging.

An important point to notice from Fig. 1 is the size at which the missing wedge becomes a problem. Some artefacts are visible even when a ±89° tilt range is used, but above approximately ±80° the effects are not too severe. With a larger missing wedge, the quality of the reconstructions deteriorates quite seriously as structures with certain orientations are no longer resolved. Typical cryo-tomograms are obtained using a ±60° range, but as discussed above, often the effective size of the missing wedge will be larger than this. The situation in these cases is likely to lie somewhere between the ±60° and ±45° panels in Fig. 1, which show that a large amount of information about the specimen will be lost. Increasing the tilt range to ±80° would clearly help this situation, while reaching ±90° would be ideal, providing a full 180° of tilt.

Several approaches have been taken to minimise or eliminate the problems of the missing wedge. Conceptually, the simplest option is to use a cylindrical specimen, which does not increase in thickness at high tilt angles and so allows image collection over a complete angular range. It is important to note that since the thickness does not change, images of a cylinder from all tilt angles have an equal S/N. Therefore even if the angular range is restricted (by mechanical limitations, for example), the effects of the missing wedge will be minimised, because images at high tilt angles will contribute useful information to the reconstruction. This stands in contrast to the situation in flat extended specimens, in which high-tilt images contribute very little to the tomogram.

Cylindrical specimens have been used in materials science, where cryogenic temperatures are not required and specimens are less sensitive to radiation. Several groups have succeeded in manufacturing cylindrical specimens for electron tomography [20–24], and modified specimen stages have been developed allowing full rotation of the specimen [24–28]. In biology, only one experiment has been reported where cylindrical specimens were used for electron tomography: glass micropipettes were used as cylindrical holders for full-tilt tomography of dry spores at room temperature [29,30], but this required the use of a high-voltage microscope and high radiation doses, and no further results have been reported since. In all these examples, the methods used would be unsuitable for frozen-hydrated specimens and so are not easily applicable to cryo-ET.

Another way to reduce the effects of the missing wedge, again predominantly used in materials science, is to make use of prior information about a specimen as a constraint on the reconstruction. In the techniques of discrete tomography [31–33] and compressed sensing electron tomography [34,35] this has allowed accurate isotropic reconstructions to be generated from very few projection images. In an extreme case, prior information allowed the reconstruction of a crystalline nanoparticle at atomic resolution from only two images [36]. Such strong prior information is not available for biological specimens at the resolutions typically reached by cryo-ET, though it is possible that future developments will allow these methods to be applied to biological specimens [35].

The only other way to reduce the effect of the missing wedge is to partially fill the empty region in Fourier space by tilting the specimen around two orthogonal axes, reducing the missing wedge to a “missing pyramid”. This technique, called dual-axis or double-tilt tomography, has been applied to frozen specimens by several groups [37–43], though it still presents technical difficulties and has not yet become routine. No doubt further developments will continue to make dual-tilt cryo-ET more accessible, but the missing region of Fourier space cannot be completely eliminated with this method and so some effects of the missing wedge will always be present. In particular, extended horizontal structures (including the slab of the specimen itself) will remain invisible in the reconstructions, and there will always be at least a small amount of elongation of compact structures.

The conclusion is that cylindrical specimens provide the only way to truly eliminate the missing wedge in cryo-ET of biological specimens. This could be critical for studies where full knowledge of a structure is essential, such as finding the complete shape of unique filamentous or membranous objects. We therefore developed a cylindrical specimen holder for frozen-hydrated biological material, based on carbon nanopipettes. These carbon tubes, with thin walls (10–50 nm) and narrow diameters (0.1–1 µm), were originally developed for electrophysiology studies [44,45] but appeared suitable for use in electron microscopy due to the low scattering contrast of their thin carbon walls.

## 2 Materials and methods

### 2.1 Manufacture of carbon nanopipettes

Glass micropipettes were prepared by drawing quartz glass capillary tubes (1.0 mm diameter) to a tip diameter of 25–35 nm using a laser-based micropipette puller (P-2000, Sutter Instrument). The micropipettes were then coated with a layer of carbon by heating to 925 °C for 1.5–2 h under a flow of methane at a pressure of 400 Torr. This process was performed using small batches of micropipettes (up to 20) placed in a porcelain combustion boat (Coorstek) and heated in a tube furnace (HST-600, Carbolite) using a work tube made of mullite (Carbolite). Argon was passed through the tube during heating and cooling, and replaced by a flow of methane (100 standard cubic centimetres per minute) when the furnace reached 925 °C. Gas flows were individually controlled by mass flow controllers (type 1479A, MKS Instruments), and the exhaust was passed to a rotary vacuum pump via an adjustable valve, allowing the working pressure inside the system to be manually controlled.

Oxygen plasma etching was carried out with a microwave-based plasma barrel etcher (MRC100, Cambridge Fluid Systems). After pumping to a low vacuum, a flow of oxygen gas (4.75 l per minute) was passed through the chamber, and microwave emission was activated for 10 s at a power of 100 W to generate the etching plasma. Glass was then removed from the tips by dipping the micropipettes into buffered hydrofluoric acid (3% HF, 17% NH_4_F) for 1–5 min.

During and after manufacture, the nanopipettes were characterised with a scanning electron microscope (LEO 1530VP, Zeiss) operated at 3.0 kV, using a secondary-electron in-lens detector and an energy-dispersive X-ray spectrometer (Inca 7426, Oxford Instruments).

### 2.2 Specimen preparation for tomography

For support in the TEM, carbon nanopipettes were attached to standard slot grids (copper, 2 mm×1 mm slot, Agar Scientific) using rapid-curing epoxy resin (Stick 2, Everbuild). This was done before specimen loading if possible, but the more complicated loading procedure used for liposomes and bacterial cells required the grids to be attached after the tubes had been filled with a liquid specimen.

Three different specimens were used in this work. (1) Ribosomes from *Thermus thermophilus* [46] were kindly provided by C. Neubauer, and mixed with 15 nm gold colloid. The expected final concentration of ribosomes was 100 nM, and of gold, 1.3×10^12^ particles/mL. (2) Liposomes were prepared from *Escherichia coli* total lipid extract (Avanti Polar Lipids). The lipids were dissolved in a buffer solution (25 mM Tris–HCl, pH 7.4, 150 mM NaCl) at a concentration of 2 mg/mL and extruded through a filter with a pore size of 0.1 µm. The liposome solution was mixed with 15 nm gold colloid (BBInternational; final concentration approximately 1.4×10^12^ particles/mL) and the membrane dye FM4-64 (Molecular Probes; final concentration 100 µg/mL). (3) *Caulobacter crescentus* cells (strain CB15N) were grown to stationary phase at 30 °C in PYE medium (0.2% Bacto peptone, 0.1% yeast extract, 1 mM MgSO_4_ and 0.5 mM CaCl_2_) with 10 µg/mL ampicillin. Cells were harvested by centrifugation and resuspended in one-tenth of their original volume in a solution of 1 mg/mL bovine serum albumin, 10 µg/ml FM4-64 membrane stain and 20 nm gold colloid (7.0×10^12^ particles/mL).

To fill a carbon nanopipette, 2–3 µL of liquid was placed in the tube and a microinjection pump was used to apply pressure to push the liquid towards the end of the nanopipette. When the liquid reached a part of the tube with a sufficiently small diameter (approximately 50–100 µm), capillary forces drew it rapidly to the tip. This process was observed either by eye or through an optical microscope (Nikon Eclipse E800 with 10× and 40× objectives and a Photometrics CoolSnap HQ2 camera). In simple cases (for example, the ribosome sample) the nanopipette was simply filled with the liquid specimen using the method just described. For larger, inhomogeneous specimens such as liposomes, where further fluid flow was required to move a target object into the correct position, a more complicated procedure was necessary. The nanopipettes were first filled with a solution of bovine serum albumin (100 µg/ml) and gold colloid (same particle size and concentration as used in the specimen solution). A standard plastic 10 µL laboratory pipette tip was filled with the specimen solution, clamped to a micromanipulator and brought to the focal point of the microscope. The nanopipette was attached to the microscope׳s specimen stage and moved towards the larger pipette tip so as to make contact with the drop of liquid. Pressure was then applied with the pump to control the flow, first drawing the specimen solution into the nanopipette and then pushing some back out if necessary.

After the nanopipette was correctly filled with the specimen, a grid was attached and the pipette shaft was broken off by gently scratching the glass surface with a diamond scribe, leaving only the pipette tip attached to the grid. This was plunge-frozen in liquid ethane, and subsequently handled in almost the same way as a normal frozen TEM grid. The only additional handling step was to rotate the grid during loading into the TEM, to ensure that the carbon tube was aligned with the microscope׳s tilt axis.

### 2.3 Electron tomography

Projection images were acquired at a range of tilt angles using an FEI Tecnai G^2^ Polara electron microscope, operating at 300 kV and equipped with a post-column energy filter and a Gatan Ultrascan 4000 CCD camera. The SerialEM software package [47] was used for automated tilt series acquisition.

All tilt series were acquired with 1° tilt increments and with the total electron dose split evenly across all images of the series. The ribosome tomogram was taken at a nominal magnification of 50,000×, with a defocus of −6 µm, zero-loss energy filtering with a slit width of 20 eV, and a total electron dose of 180 e^−^/Å^2^. For the liposome tomogram conditions were the same, except that the nominal magnification was 41,000×, the defocus was set to −10 µm, and the total electron dose was 150 e^−^/Å^2^. The tomogram of *C. crescentus* cells was collected at a magnification of 22,500×, with a defocus of −6 µm, a total electron dose of 110 e^−^/Å^2^, and no energy filtering.

The tilt series were aligned and processed using the IMOD software package [48], and reconstructions were generated using the SIRT algorithm implemented in Tomo3D [49]. Anisotropic nonlinear diffusion was carried out using TomoAND [50,51]. Line profiles were generated using ImageJ [52], and volume rendering of tomograms was performed using PyMOL [53].

## 3 Results

### 3.1 Manufacture of carbon nanopipettes

**Fig. 3:**
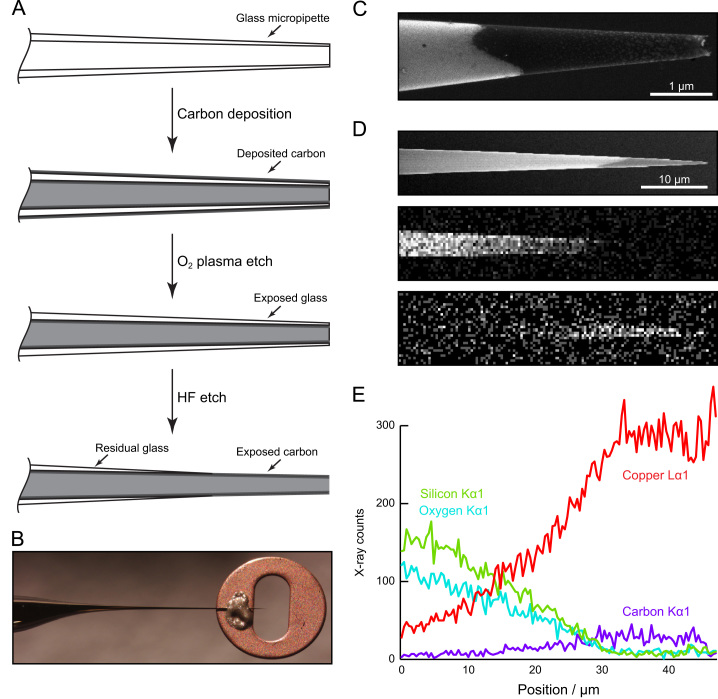
Carbon nanopipette (CNP) manufacture and characterisation. (A) Schematic of the steps used to fabricate carbon nanopipettes. (B) Photograph of a CNP attached to a slot grid. (C) Scanning electron micrograph of the tip of a finished CNP, showing a clear contrast between the residual glass and the exposed carbon. (D) Reference SEM image (top) and 2D EDX maps of X-ray emissions from silicon (middle) and carbon (bottom). These show that the observed image contrast in the SEM matches the positions of the glass and carbon recorded by the EDX detector. (E) EDX linescan showing signals recorded for four elements along the tube shown in part D. The silicon and oxygen signals show the glass thinning and disappearing towards the tube tip, where the carbon layer beneath is revealed. The copper signal comes from the background material behind the tube, and indicates the tube׳s electron transparency.

Carbon nanopipettes were made using an adaptation of previously-published methods [44,45,54,55]. More information on the development of the manufacturing process is available in Ref. [56]. Briefly, quartz glass micropipettes were used as templates for the deposition of a layer of carbon by chemical vapour deposition from methane at 925 °C. The carbon on the outer surface of the pipettes was removed by oxygen plasma etching, leaving glass tubes with a carbon coating on the inner surface only. The glass layer was subsequently removed from the pipette tips with hydrofluoric acid, exposing a few micrometres of the carbon layer from the inside of the tubes. A schematic diagram of this manufacturing process is shown in Fig. 3A.

During and after manufacture, the carbon nanopipettes were characterised by scanning electron microscopy (Fig. 3C). Energy-dispersive X-ray spectroscopy was used in both mapping and line scan modes to examine the chemical composition of the nanopipettes, confirming that glass was absent from the tips and electrons could pass through the carbon to excite X-ray emission from the copper background (Fig. 3D and E). Tip diameters were measured using the SEM, allowing suitable pipettes to be selected for tomography experiments. Typical tip diameters after manufacture were around 400–800 nm, and the carbon wall thickness was in the range of 10–20 nm.

### 3.2 Ribosomes

Three frozen-hydrated specimens were examined inside cylindrical holders by electron tomography. Ribosomes were used as the first test specimen because they can be prepared at high concentrations, and are very small relative to the dimensions of the tubes, allowing a ribosome solution to be treated as effectively homogeneous for these experiments. This allowed a relatively simple specimen loading method to be used, in which the specimen solution was placed inside the nanopipette and then pushed to the end. Since the specimen was effectively homogeneous, it was reasonably certain that if any liquid reached the tip then some of the specimen would be present. This method avoided too much disturbance of the delicate carbon-walled section at the end of the nanopipette, reducing the rate of tip breakage.

**Fig. 4:**
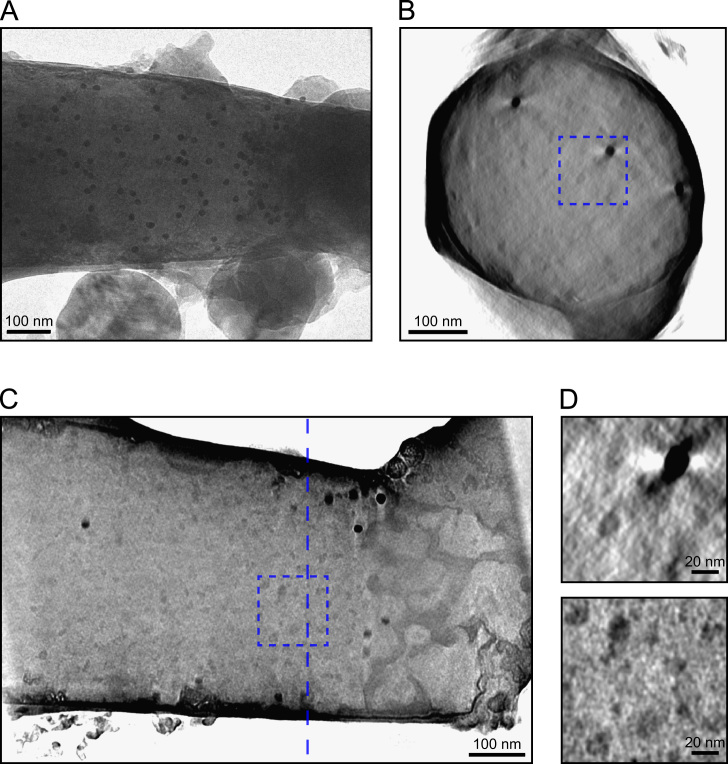
Tomography of ribosomes. (A) One image from the tilt series. Crystals of ice contamination are visible outside the tube, while 15 nm gold particles can be seen inside. (B) A cross section from the reconstruction. (C) A slice in the *xy* plane of the reconstruction. The vertical dashed line marks the position of the cross section shown in part B. (D) Magnified views of the areas marked by dashed boxes in parts B and C. Ribosomes and a gold particle are visible.

For the most successful ribosome tomogram, a carbon nanopipette with a tip diameter of approximately 400 nm was attached to a slot grid, filled with a solution of ribosomes and 15 nm gold particles, and plunge frozen. A tilt series was collected over an angular range of ±73° (Fig. 4A) and used for tomographic reconstruction (Fig. 4B–D). Gold particles are clearly visible in the reconstruction, along with a larger number of similarly-sized, less dense particles, which are likely to be ribosomes or dissociated ribosomal subunits. The approximate diameter of the tube is 450 nm when measured at approximately 0.5 µm from the tip. This is comparable to the initial 400 nm size of the tube, so it is likely that the tube tip was not damaged during preparation of the specimen.

Some images in the tilt series displayed a mottled or striped pattern (not shown), indicating that the ice inside the tube was crystalline. There were also several large ice crystals attached to the outside of the tube. Despite these imperfections, the quality of the tomogram is reasonably good, allowing the ribosome particles to be seen. Some effects of the missing wedge are visible: there are white halos around the gold particles, and the tube walls are not quite resolved in the *z* direction. However, these effects are much reduced compared to typical cryo-tomograms (compare Figs. 4B with 2). An attempt was made to average the ribosome particles together to confirm their identity, however no distinctive features were recovered and the averaged map remained roughly spherical, indicating that there is insufficient signal in the particles to allow proper alignment (data not shown).

### 3.3 Liposomes

Larger specimens – such as liposomes, virus particles and bacterial cells – could not be assumed to be homogeneous in solution, and so had to be specifically positioned in the narrowest part of the tube tip where they would be visible for tomography. This required a more complicated loading process, in which liposomes were stained with fluorescent dye and manipulated under a fluorescence microscope. The simplest way to move liposomes to the tube tip would be to fill the tube with a solution of liposomes and then push liquid out of the tip until a liposome reaches the desired position. However, it was impossible to do this with the tube tip in air, due to the high surface tension of the liquid at the end of the tube. To break this surface tension, the tube was dipped into another drop of liquid. This allowed fluid flow through the tube tip in both directions, under the control of a microinjection pump. Despite trying a number of different microscope configurations, it proved very difficult to observe the tube tip continuously while the liquid was moved through it (which would have been the ideal way to allow the process to be stopped when the specimen reached the correct position). The most successful method was found to be to dip the tube into a liquid drop suspended on the tip of a pipette, guided by observation through the microscope at low magnification. The pipette and liquid drop obscured the tube tip, preventing imaging of the tube while the liquid flow was occurring. However, by repeated cycles of dipping and withdrawal, it was possible to induce liquid flow in short bursts and make occasional observations to guide the process, allowing liquid flow to be stopped at roughly the correct point. This method was substantially more complicated than the simple filling procedure described in the previous section, and many tubes were destroyed by mishandling, or were found to be broken after loading.

**Fig. 5:**
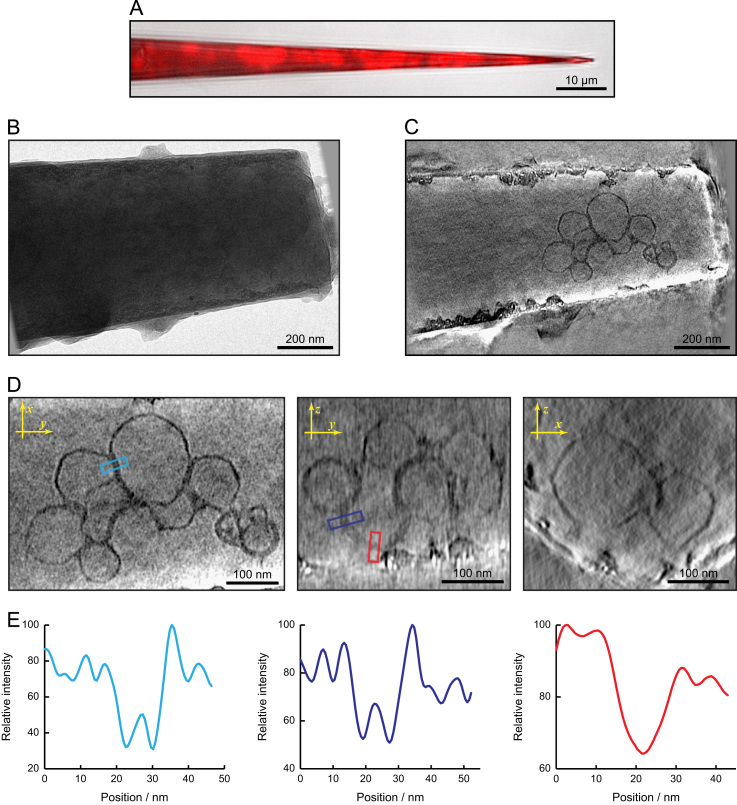
Tomography of liposomes. (A) Overlaid optical images of the filled carbon nanopipette. The tube shape can be seen in the brightfield transmitted light image (greyscale) and the fluorescence image from the FM4-64 membrane dye is shown in red. (B) An image from the TEM tilt series. A few gold particles are visible. (C) A slice from the reconstruction. A group of liposomes is clearly visible. (D) Three orthogonal slices, showing the visibility of the liposome membranes in almost all orientations in the tomogram. (E) Line profiles (averaged across a 10.5 nm width and 12 nm slice thickness) for the areas marked by coloured boxes in part C. A pair of minima shows that two membranes at a spacing of 7.5 nm can be resolved in the *xy* plane, but the pair is not resolved in the *z* direction. (For interpretation of the references to color in this figure legend, the reader is referred to the web version of this article).

Experiments showed that filling a carbon nanopipette with a liposome solution and then pushing the liquid out of the end was generally unsuccessful, because the liposomes adhered to the carbon walls of the tube and did not reach the tip. Addition of bovine serum albumin to the solution helped reduce the severity of this problem but did not eliminate it. More successfully, nanopipettes were filled with a solution containing only BSA and gold particles, then dipped into a solution of liposomes. A small amount of the liposome solution was sucked into the end of the tube, after which liposomes were observed inside the tube, though usually not at the tip itself. Subsequent dipping back into the drop of liposome solution, combined with slight pressure to push liquid out of the tip, succeeded in leaving some liposomes in the end of the tube (Fig. 5A). After observation, tubes that were filled correctly were attached to EM grids and plunge frozen.

From these tubes, one was found which had relatively little surface ice contamination and a sufficiently small diameter for TEM imaging (approximately 550 nm). A tilt series was collected from this tube over an angular range of ±72.5° (Fig. 5B). As with the tube containing ribosomes, the ice inside the tube was observed to be crystalline. Gold particles are visible in the projection images, but liposomes cannot be seen. Some liposomes were visible in an initial tomographic reconstruction, but the contrast was low. To remove any adverse effects from the extreme contrast difference between the tube and its surroundings, the tilt series was processed to replace the high-intensity background outside the tube with a flat grey of the same level as the mean inside the tube. After reconstruction, liposomes could be seen inside the tube with good contrast, though the tube edges displayed some artefacts (Fig. 5C), notably a band of high brightness near the tube walls, and the disappearance of the carbon in some places. The high brightness was caused by the thinner ice at the edge of the tube, which was brighter than the mean value used to replace the background, while some of the carbon was removed along with the background during image processing.

Slices taken from orthogonal directions within the tomogram showed that liposome membranes could be seen in all orientations, albeit more weakly in the *z* direction (Fig. 5D). In some places, closely-spaced pairs of membranes were observed. Averaged line profiles from these areas allow the spacing between them to be measured at about 7.5 nm (Fig. 5E), which provides an estimate for the resolution of the tomogram in the *xy* plane. The pair of membranes is not resolved in the *z* direction, indicating the effect of the missing wedge.

**Fig. 6:**
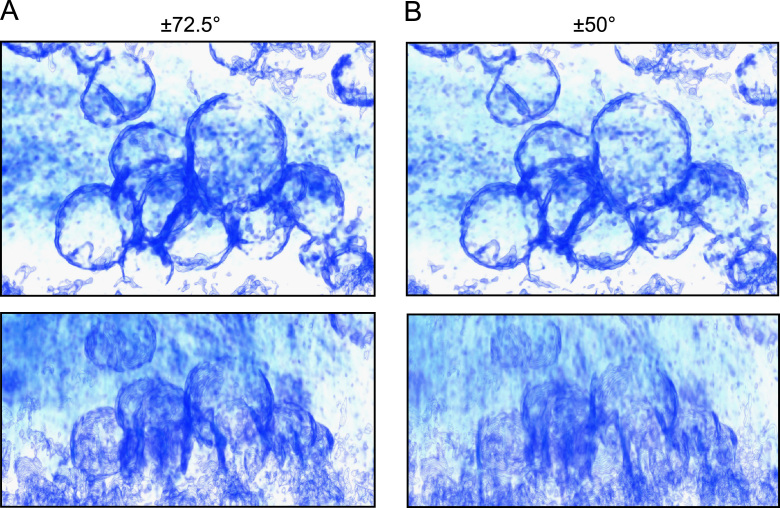
Static volume-rendered views of the liposome tomogram. (A) Two views of the tomogram reconstructed from the full tilt series (angular range ±72.5°). Top: view along the *z* axis. Bottom: oblique view roughly intermediate between the *x* and *z* axes. (B) Equivalent views of a tomogram reconstructed from only part of the tilt series (angular range ±50°), demonstrating the degradation caused by the larger missing wedge.

Finally, volume rendering was used to illustrate the improved three-dimensional appearance of tomograms collected over such a large angular range. Two tomograms were generated, one using the full tilt series, and the other using only the images from an angular range of ±50° to simulate the missing wedge size of a typical cryo-tomogram from a slab specimen. Both tomograms were filtered using anisotropic nonlinear diffusion to reduce noise [50,57] and visualised by volume rendering as shown in Video 1 and Fig. 6. Several groups of liposomes can be seen inside the tube, with an appearance reminiscent of soap bubbles. Viewing along the *z* axis (Fig. 6, top), both tomograms look similar, but an oblique view (Fig. 6, bottom) reveals that the vesicles appear fully closed and “three dimensional” in the ±72.5° tomogram, while in the ±50° reconstruction the vesicles appear incomplete.

A range of ±50° was thought to provide a fair comparison since the tube thickness does not change with tilt angle, and images from a cylinder at all tilt angles have the same S/N. As discussed in the Introduction, in a typical cryo-tomogram collected over a ±60° range, the S/N of the images is gradually reduced as the tilt angle and effective specimen thickness increase. The effect of the missing wedge is therefore greater than would be expected from the tilt range. Using the angle at which cell membranes become invisible as an indicator of the effective missing wedge size (Fig. 2), a ±50° range of images with equal S/N should be roughly equivalent to a tomogram from a ±60° range with S/N decreasing at high tilt angles.

### 3.4 Bacterial cells

Being relatively large objects, bacterial cells generally required the same loading procedure as liposomes (that is, dipping the tube to break surface tension at the tip, then moving the liquid to try and position a target object in the correct place at the extreme end of the tube). With bacteria this was made more difficult because the size of the cells closely matched the available space in the tube. They were therefore more prone to blocking the tube, or simply failing to move to the correct position at the tube tip. The difficulty depended strongly on the size of the tubes: in larger tubes (greater than 1 µm in diameter) it was relatively simple to position cells correctly in the tip, but these were then too thick for TEM imaging at 300 kV. With smaller tubes, loading was more difficult and many tubes were broken during the loading process. Cells were never observed in the correct position in the extreme tip of any of the tubes.

**Fig. 7:**
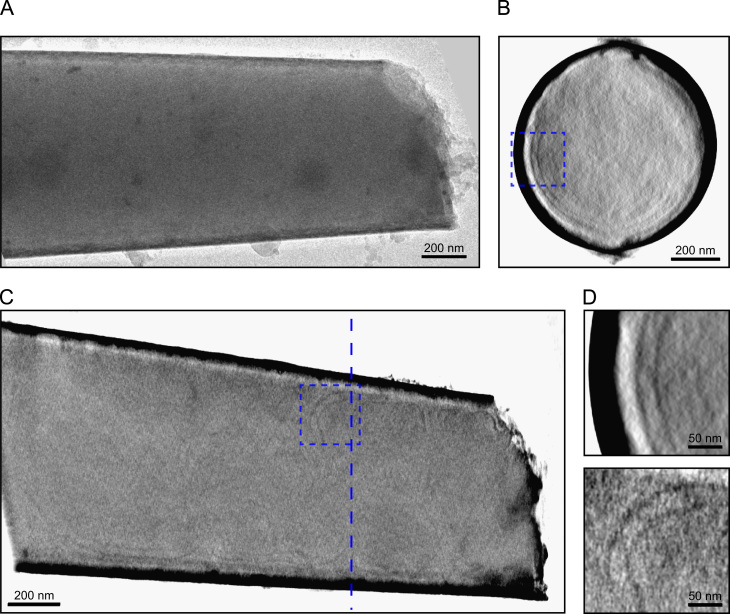
Tomography of bacterial cells. (A) An image from the tilt series. Some gold particles are visible along with a small amount of surface ice contamination. (B) A cross section from the reconstruction. (C) A slice in the *xy* plane of the reconstruction. The vertical dashed line marks the position of the cross section shown in part B. (D) Magnified views of the areas marked by dashed boxes in parts B and C. Paired, extended features are visible, likely to be cell membranes.

The only successful attempt to collect a tomogram of bacterial cells began with a tube approximately 450 nm in diameter. After simple loading with a culture of *Caulobacter crescentus* cells (without dipping the nanopipette), some bacteria were observed by fluorescence at a distance of a few micrometres from the tube tip, with none visible in the tip itself. After freezing and loading into the TEM, the tube diameter was measured to be 830 nm, indicating that the end of the tube had broken off. This had the effect of bringing the end of the tube back to roughly the position where the cells had been observed. A tilt series was collected from this tube over an angular range of ±79° (Fig. 7A). No signs of crystalline ice were seen in the images from this tilt series, in contrast to the ribosome and liposome specimens discussed previously. (This was typical for the cylindrical specimens tested in this work. Overall, approximately half of our specimens showed signs of crystalline ice inside the tube, and the other half did not, over a range of tube diameters up to 1.1 µm.)

No complete cells could be seen in the reconstructed tomogram, but near the edges of the tube, several paired linear features were observed (Fig. 7B–D). Image features would be expected to be most visible near the tube walls where the ice thickness is lowest, and a previous report confirms this effect [58]. These linear features appear to be separated by a constant distance of approximately 30–35 nm, consistent with previous tomographic observations of the double membrane envelopes of *C. crescentus* cells [59]. The lines are continuous over several hundred nanometres, are straight or smoothly curved and do not cross each other. It therefore seems reasonable to suggest that they are cell membranes, and so this tube did indeed contain bacterial cells. The remainder of the cell envelopes and internal features cannot be seen, due to the decrease in observed contrast in the middle of the tube caused by the greater ice thickness.

## 4 Discussion

These results demonstrate that it is possible to obtain electron tomograms of frozen-hydrated biological material inside cylindrical holders. This is the first time that tomograms from frozen cylindrical specimens have been reported. In principle, it would be possible to use these to collect tilt series over a complete angular range of 180° and therefore completely eliminate the missing wedge. However, this is currently prevented by technological limitations of the microscopes. In particular, a full-tilt goniometer capable of maintaining cryogenic temperatures would need to be developed. Various full-tilt specimen stages have been developed for applications in materials science [24,25,28], but none of these would be easily adapted for cryogenic operation and it is likely a new design would be needed.

Cryo-tomography inside cylindrical carbon tubes has several advantages. Most obviously, it is possible to reduce or eliminate the missing wedge, with all of the resulting benefits for isotropic resolution and completeness of information about the specimen. The importance of this can be seen by returning to Fig. 1. A comparison of the original phantom image with the ±60° reconstruction shows how much a typical cryo-tomogram (collected over a ±60° range) would be degraded by the missing wedge. The ±90° panel shows the enormous improvement which could be gained by full tilting of a cylindrical specimen, and the ±80° panel shows the theoretical quality which could be achieved even using existing microscope systems, where tilting close to ±80° is possible in some cases (for example, the bacterial cell specimen presented here, which was tilted to ±79°).

Also important is the improved control over specimen thickness and composition. The tube thickness can be directly measured in single TEM images, without resorting to indirect methods such as cutting holes with the beam or inferring the ice thickness from beam intensity measurements. Evaporation from the specimen is almost eliminated by the surface layer of carbon, meaning that biological objects will be undisturbed by the effects of increasing solute concentration or constriction within a thinning layer of water during freezing. The avoidance of constriction in the water layer also means that large objects such as bacterial cells will not be squashed flat. This slightly increases their thickness but prevents any distortion of cellular structures, which could be a significant advantage for certain studies.

Opposing the advantages just discussed, the use of cylindrical holders for cryo-tomography suffers from some limitations. Specimen preparation remains the major challenge, for two reasons: firstly, the carbon tubes used in this work are fragile and easily broken, and secondly, correctly positioning a specimen of interest in the usable narrow section at the end of a tube is quite difficult. Improvements in the procedures used for optical microscopy and specimen loading will probably be necessary for this method to be usefully applied to structural biology.

Given the relative ease of specimen loading in tubes with larger diameters (on the order of a few micrometres), the most promising approach might be to combine this method with focussed ion beam (FIB) milling to remove excess ice and carbon from the areas around the target specimen. This would combine the advantages of both methods, allowing the easy positioning of a desired specimen (selected by fluorescence microscopy, for example) inside a capillary that would provide protection from distortion and evaporation during the freezing process. Subsequent thinning with the FIB would reduce the ice thickness to allow high-quality TEM imaging, while avoiding both the challenge of finding the target in a large extended sample and the need to mill away large amounts of material, which currently hinder existing approaches to specimen preparation by FIB milling.

## Figures and Tables

**Video 1:**
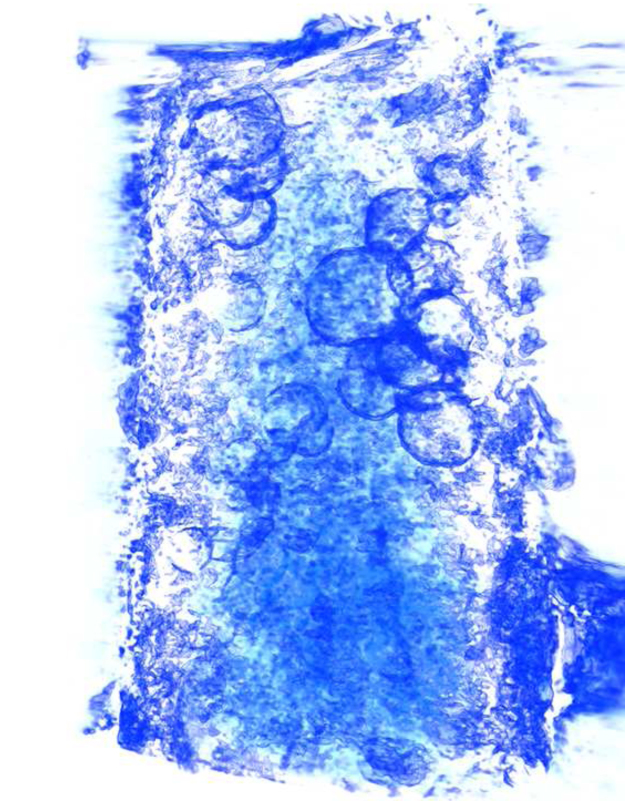
Volume rendering of the liposome tomogram. Several groups of liposomes can be seen inside the tube. Most of the outer wall of the tube has been removed during image processing, but some signs of rough features on the surface can still be seen. A video clip is available online. Supplementary material related to this article can be found online at 10.1016/j.ultramic.2013.10.016.
